# The Importance of Pine Species in the Ethnomedicine of Transylvania (Romania)

**DOI:** 10.3390/plants11182331

**Published:** 2022-09-06

**Authors:** Nóra Papp, Dragica Purger, Szilvia Czigle, Dóra Czégényi, Szilvia Stranczinger, Mónika Tóth, Tünde Dénes, Marianna Kocsis, Anna Takácsi-Nagy, Rita Filep

**Affiliations:** 1 Department of Pharmacognosy, Faculty of Pharmacy, University of Pécs, 7624 Pecs, Hungary; 2 Department of Pharmacognosy and Botany, Faculty of Pharmacy, Comenius University Bratislava, Odbojárov 10, 832-32 Bratislava, Slovakia; 3 Department of Hungarian Ethnography and Anthropology, Babeş-Bolyai University of Cluj-Napoca, 400202 Cluj-Napoca, Romania; 4 Department of Plant Biology, Faculty of Sciences, University of Pécs, 7624 Pecs, Hungary; 5 Institute of Pharmaceutical Technology and Biopharmacy, Faculty of Pharmacy, University of Pécs, 7624 Pecs, Hungary

**Keywords:** ethnobotany, Hungarians, *Abies alba*, *Picea abies*, *Pinus* spp.

## Abstract

The geographical and ecological features of Transylvania enable the wide ethnobotanical use of pine species. The aim of this study was to survey the current ethnomedicinal and other traditional use of pine species of Hungarian-speaking ethnic groups in Transylvania and to compare them with earlier reports performed in Transylvania and from other countries related to the Carpathian Basin. Information on pine species was obtained using semi-structured interviews with 515 Transylvanian informants from 18 villages in the period 2007–2019. The young shoots of *Abies alba* Mill., *Picea abies* (L.) H. Karst., *Pinus nigra* J. F. Arnold, and *Pinus sylvestris* L. were applied to treat respiratory diseases, while the resin was used for dental problems. Syrup and decoction were made from the cones of all species, except *Abies alba*. *Picea abies* was the most frequently documented with seven preparations from different parts (even needles), and this species was mentioned in the treatment of 21 diseases. The least recorded was *Abies alba*, which was applied for coughs and decayed teeth. We recorded the use of the cones and needles of *Picea abies* for dyspnoea, thyroid glands, and kidney disorders, previously unknown in ethnomedicinal literature. Our data on the pine species confirm their current use and significance in Transylvania.

## 1. Introduction

The pine family (Pinaceae) is among the largest plant families of conifers, comprising 11 genera including altogether 225 species [1]. They are native to the northern temperate regions of the Earth [2]. According to historical records, pine species, such as *Abies alba* Mill., *Picea abies* (L.) H. Karst., *Pinus nigra* J. F. Arnold, and *Pinus sylvestris* L. are widely used in the world for different purposes.

Throughout human history, pine species have been used in traditional medicine for various ailments [3]. Ethnobotanical surveys carried out in different countries of Europe reported several medical uses of pine species, e.g., in the treatment of respiratory problems (e.g., colds, coughs) [4,5], skin diseases (e.g., abscesses, furuncles, and wounds) [6,7,8], and are also used as a vitamin source [9].

The knowledge and experience about the medicinal use of species from the *Abies*, *Picea*, and *Pinus* genera were gained over centuries and reported in written form in the official materia medica. In the work of Matthioli [10], the resin and oleum of *Abies* spp. and *Picea abies* have been regarded as curative emplastrum for wounds, verrucas, and as diuretics in the adjuvant treatment of minor urinary complaints. The cones, bark, seeds, and resin of *Pinus* spp. have been applied as *Vinum medicinalum* for fevers, colds, coughs, tuberculosis, urinary inflammation, toothaches, furuncles, as well as externally as emplastrum for wounds, blaze, burns, and lachrymation [10]. The resin of *Picea abies* has been described as emplastrum and unguentum to treat skin diseases and inhalation in coughs associated with the common cold [11].

The data about the medicinal use of pine species have appeared in the *Extra Pharmacopoeia*, which gave details of *Oleum therebinthinae* and *Pinus sylvestris* in galenical formulations, such as enema, emulsion, liniment, and *Linimentum terebinthinae aceticum* (as an antirheumatic agent). The drugs are recommended for fever, lumbago, rheumatism, and as a mild stimulant inhalation in chronic laryngitis [12].

In the national pharmacopoeias of the Habsburg Empire and Austro-Hungarian Monarchy issued between the second half of the 18th century and the beginning of the 20th century, several official drugs were derived from *Pinus sylvestris* [13,14,15,16,17]. In the *Pharmacopoeia Hungarica* (editions I–VIII, 1871–2006) [18,19,20,21,22,23,24,25], the drugs of the *Picea*, *Pinus*, and *Larix* genera of the Pinaceae family were mentioned: resin (e.g., *Emplastrum adhaesivum borussicum*), essential oil (e.g., *Oleum pini sylvestris pro inhalatione*), and balsam of turpentine and turpentine oil (e.g., *Terebinthinae communis liquatae et colatae*). In the last (10th) edition of *Romanian Pharmacopoeia*, the volatile oil of *Pinus mugo* (*Pini montanae aetheroleum*) was discussed for its antiseptic effect [26]. At the beginning of the 21st century, the therapeutic significance of pine species gradually vanished, and currently, only *Pinus sylvestris aetheroleum* is officially listed in *The European Pharmacopoeia* [27].

A growing number of studies suggest that pine species have an important role in medicine. They have been investigated for antioxidant [28,29,30,31], anti-inflammatory [32,33,34], antibacterial [29,35], antiviral [36], anticancer, and cytotoxic activities [37,38,39]. Yang et al. [40] suggest that the crude extracts and some chemical constituents of the *Abies* species were found to possess antibacterial, antifungal, anti-inflammatory, and antitussive activities. The species of the genus *Pinus* are reported for their antimicrobial, antioxidant, anti-inflammatory, and cytoprotective properties [41,42]. They can also be used in the treatment of neurodegenerative disorders, such as Alzheimer’s and Parkinson’s diseases [41,43]. The antimicrobial and antioxidant activities of the *Picea* species extracts were confirmed in several studies [44,45,46].

Among the most widely dispersed species in Europe, *Abies alba* has been mainly used for ships, painter and writing boards, and carpentry. In Ancient Rome, the branches of *Picea abies* laid on the threshold of houses were considered a symbol of grief. Additionally, in Antiquity, cork and lignum of *Pinus nigra* and *Pinus sylvestris* were utilized as timber and for container manufacturing and baskets for cereal and grape transport, while the fresh cork was used as paper [47].

Traditional plant use has played a significant role in Transylvania (North-West Romania) [48]. Due to their isolation and insufficiency of official medical care, the inhabitants of several Transylvanian villages have valuable archaic knowledge of plants [49,50]. Comprehensive ethnobotanical research was carried out in the second part of the 20th century, mainly focusing on isolated settlements [51,52,53,54,55,56,57,58]. The recent ethnobotanical surveys focus primarily on the maintenance of the traditional harvesting methods, applied drugs, ethnomedicinal use of plants, and treating disorders in human and veterinary medicine [59,60,61,62,63,64,65]. Currently, the decline in the implementation of the traditional use of medicinal plants is caused by the alteration and degradation of the environment and more expanded availability of official medicines and modern pharmaceuticals in several regions of Romania. Moreover, the transmission of traditional knowledge from elderly people is also declining due to increased migration of the young generation to larger cities or abroad [64]. Nevertheless, several ethnic groups still preserve their ethnomedicinal heritage through home practices and oral transmission of their knowledge. In this study, we sought to obtain a better understanding of ethnobotanical knowledge related to the use of pine species in Transylvania. We organized our research around three objectives: (1) to reveal the current local name of pine species and their plant parts; (2) to document current ethnobotanical use of pine species; (3) to compare our records with previous data from Transylvania and other Central and South European countries related to the Carpathian Basin to make known the importance of pine species. Based on the landscape and vegetation features of the study area, our focus was on *Abies alba*, *Picea abies*, *Pinus nigra*, and *Pinus sylvestris*.

## 2. Results

In our study, 515 informants were interviewed, and approx. 70% of them shared their ethnobotanical knowledge about pine species (*Abies alba*, *Picea abies*, *Pinus nigra*, and *Pinus sylvestris*). Among the interviewed persons, the ratio of women to men was 2:1. The average age was 65 years.

### 2.1. Local Terminology of the Species and Used Plant Parts

To identify the pine species, informants used the vernacular description of the color, the tip and the length of the needles, the color of the bark, the position of young shoots and branches, and the shapes and features of the cones. In this respect, the inhabitants of Martiniş and Petreni described the needles of *Abies alba* as being ***white*** and ***blunt*** (without stinging tip). In Martiniş the young shoots of *Abies alba* are referred to as a ***candle***. The informants from Lueta depicted the spiky stinging needles of *Picea abies* as reddish in summer. In Chinușu, Martiniş, and Petreni, *Pinus nigra* was said to have the longest needles, while in Martiniş, *Pinus sylvestris* was characterized as having shorter ***pale green*** leaves. In Lueta, the branches of *Picea abies* were described as ***diversified***, while the trunk of *Pinus sylvestris* was described as ***winding*** and the bark as ***weak*** and ***red***.

The number of local names in the study area varied from four to seven per species. Unique and overlapping names occurred among the local names of the pine species (Table 1). For example, ***vörösfenyő*** was used both for *Picea abies* (in four villages) and *Pinus sylvestris* (in six villages). However, it is the official Hungarian name of *Larix decidua* Mill., which is not discussed in this study. The greatest overlapping was observed between *Pinus nigra* and *Pinus sylvestris*, alternatively referred to as ***luc***, ***lucfenyő***, and ***lukszfenyő***, even though in Hungarian plant terminology, *lucfenyő* is the official name of *Picea abies*.

Nevertheless, the correct identification of each selected species was performed based on the morphological descriptions given by the informants and upon collected voucher specimens. The local names of the young fresh shoots of each species were ***újulás***, usually collected in early spring. The resin of each species was named ***szurok***. ***Fenyőalma*** (in two villages), ***fenyőtubus*** (in one village), ***buba*** and ***fenyőbuba*** (in one village), ***csusza*** (in one village), and ***csalóka*** (in five villages) were documented as mostly denominating the young, fresh, and red female inflorescence also harvested in spring. The needles are known as ***fenyőcserhe*** or ***tövis*** (one and one village, respectively).

### 2.2. Ethnomedicinal Uses

*Abies alba*, *Picea abies*, *Pinus nigra*, and *Pinus sylvestris* were applied in the ethnomedicinal practice in the study area (Table 2). The frequency of the recorded data about selected species differed. *Picea abies* was the most frequently documented; it was mentioned in the treatment of 21 diseases. The young shoots of all pine species were used to treat respiratory diseases, while the resin of all species was applied for dental problems. The cones of all species were used, except *Abies alba*. The least recorded was *Abies alba*, used only for coughs and decayed teeth (Table 2).

The preparations of drug parts varied between two and seven per species, and most preparations (seven) were used from different parts of *Picea abies*. The use of needles was recorded only in the case of *Picea abies*, while the cones and resin of each pine species were documented as food in various forms (Table 2).

The bark, cones, needles, resin, and shoots of *Picea abies* were used in different preparations for medicinal purposes. The decoction of the bark was applied in the treatment of varicose veins in Lueta. Syrup and decoction were made from the cones and used internally or externally. The decoction of one bushel (−35 L) of cones was prepared with 40 L of water and applied for rheumatic pain (e.g., backache). The young cones of *Picea abies* were collected in the flowering period and boiled with sugar as a sweet named ***dulcsáca***, similar to honey, and added to desserts by teaspoon. The decoction of the needles, prepared by soaking one teaspoon of the leaves in 1 L of water, was applied for urinary bladder disorders (e.g., cystitis) in Lueta. The resin can be collected from the trunk in dried form or by letting it run in a bottle. It is applied for skin injuries and furuncles as an ointment (in Lueta) and for backache as a poultice (in Ghipeș). The melted resin was mixed with oil and incorporated into ointments to treat furuncles (in Porumbeşti) and wounds, furuncles, and tooth decay (in Călugăreni). In complex preparations, the resin was generally mentioned as a stiffening agent; it is furthermore an excipient that facilitates the drying process and gives a unique fragrance to homemade ointments. In the treatment of hand skin diseases, the resin of *Picea abies* combined with six or eight other components was used in ***7-ír*** (= seven-part lotion) and ***9-ír*** (= nine-part lotion) and also with the leaf of ***útilapi*** (*Plantago lanceolata* L.) as sticking material for a poultice. In the ointment formulations, 100 g of resin was usually mixed with 2 dL of oil or 100 g of wax, and tallow pork fat was used to treat furuncles. As a poultice for treating furuncles, beeswax and oil were incorporated into the formulation (in Lueta). The young shoots of the species collected in spring were soaked for a day in water in a 1:1 ratio and then boiled with sugar; the brew is then filtered, dry-steamed, and stored in a warm place until cooled. Two or three teaspoons or one cup daily of this syrup was used for asthma in children and adults (in Lueta, Lunca de Sus, and Martiniş).

*Picea abies* was recorded for treating respiratory disorders as a green or red syrup prepared from the young shoots or the cones mixed with honey or sugar (for taste enhancement and preservation). The syrup made from this species contained the young shoot (200 g), sugar (650 g), and water (350 g) boiled and applied for respiratory problems (in Turulung). Both the decoction and the syrup of the young shoots were documented in Lueta for circulatory issues and as sedative remedies. The old branches of *Picea abies* were used for several multiherbal remedies in Lueta, e.g., (1) boiled with the bark of ***bojzafa*** (*Sambucus nigra* L.) for the treatment of hemorrhoids; (2) boiled with the bark of ***fűzfa*** (*Salix alba* L.) and ***cserefa*** (*Quercus* spp.) and mixed with one handful of salt and one teaspoon of potash alum to treat varicose veins of the legs; and (3) boiled with the leaves of *Fragaria vesca* L., *Rubus idaeus* L., *R. caesius* L., and *Juglans regia* L. and the pseudofruit of ***szaragógya*** (*Rosa canina* L.) to treat paralyses and also used as a bath. For thyroid disorders, five handfuls of branches are boiled in 5 L of water and applied as a poultice in the evenings and as a gargle during the day. Honey of this species alone was used for stomatitis in Lueta.

**Figure 1:**
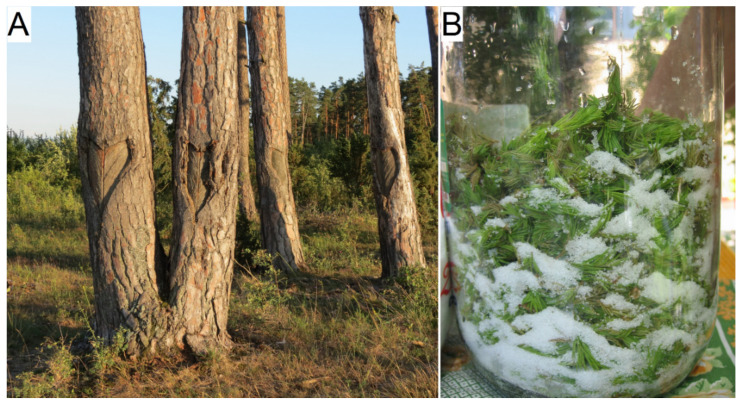
(**A**) Resin collection by tapping the trunk (in Petreni) and (**B**) Preparation of syrup from the young shoots (in Lueta).

The cones, resin, and shoots of *Pinus nigra* were used in different preparations for medicinal purposes in the study area (Table 2). The resin was used in the treatment of skin disorders and decayed teeth. The resin was collected in Petreni by tapping the trunk in a V-shape, heart-shape, or wedge-shape and letting the resin flow out into a pot through a metal spout and then using it for the treatment of wounds (Figure 1A).

The young shoots of *Pinus sylvestris* were used to treat respiratory diseases (e.g., asthma, coughs, and tracheitis). They were collected in spring, cut at about 10 cm from the end of the branches, and used for preparation: one kg of shoots is soaked in 1–1.5 L of water, boiled slowly until the shoots have softened, mixed with citric acid to extract the taste, soaked for 1–2 days, filtered through gauze, and then mixed with sugar with the ratio of sugar and liquid being 1:1 (in Chinușu, Orășeni, and Petreni). This syrup, whose color is red, often is added (1–2 drops) in herbal tea (in Chinușu). In other prescriptions, shoots can be soaked in honey for 3 days or layered with sugar in a bottle for 2–3 months (in Martiniş) (Figure 1B). In this village, people inhale the resin-like fragrance of the fresh shoots against cold.

The resin from a matchstick was melted above a candle and blended with tallow in the treatment of wounds caused by the dryness of the hands (in Orășeni), with honey for furuncles (in Mereşti), or applied directly onto decayed teeth (in Mereşti and Sânpaul). A long time ago, children chewed the resin picked from the trunk as a dessert and tooth cleaner (in Lueta and Martiniş). The alcoholic extract (brandy) of the resin of *Pinus sylvestris* is known as ***esszenc*** (in Martiniş). The honey made by bees in pine forests is also consumed in tea (in Lueta).

**Figure 2:**
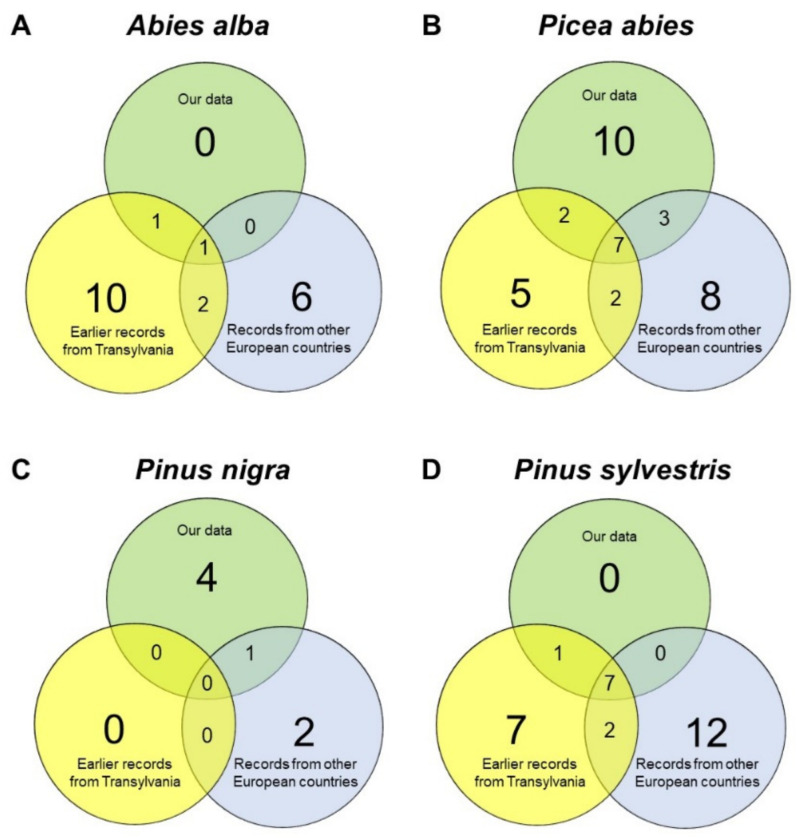
Venn diagram showing the total number and overlapping of medical uses recorded in the study area and other countries related to the Carpathian Basin. (**A**) *Abies alba*, (**B**) *Picea abies*, (**C**) *Pinus nigra*, and (**D**) *Pinus sylvestris*.

Comparing our records to previous data from Transylvania and other countries related to the Carpathian Basin, it is obvious that we cannot confirm several previously described medicinal uses of *Abies alba* and *Pinus sylvestris*, nor did we register any new applications. In contrast, for *Picea abies* and *Pinus nigra*, we recorded lots of new data that were not previously known (Figure 2).

### 2.3. Other Uses

In addition to the ethnomedicinal use, the studied pine species were mentioned as being used for various other purposes (Table 3).

**Figure 3:**
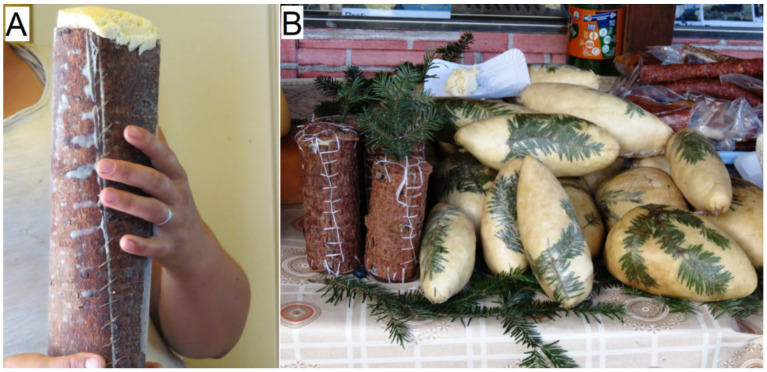
(**A**) Cottage cheese container made of bark of pine species (in Cinod) and (**B**) Pine needles for cheese conservation.

The decoction of the bark of *Picea abies* boiled with the bark of *Quercus* spp. was applied to tan raw leather. The bark of *Abies alba* was used for shoe soles, while the bark of *Pinus sylvestris* and *Pinus nigra* was employed to make cottage cheese containers (Figure 3A).

The cones of *Pinus sylvestris* and *Pinus nigra* were described as a tinder material. The resin of pine species is used for various purposes: the resin of *Picea abies* and *Pinus sylvestris* was used, e.g., for rosining violin bows, that of *Pinus nigra* as adhesive and dye additive, while the resin of *Picea abies* was utilized for homemade soap, human epilation, and hair removal during pig slaughter. Before slaughtering pigs, the inhabitants collected the previously stove-dried and ground resin of *Picea abies*; they scattered it on the animal, adding hot water to form a balm to accelerate the removal of the bristles. The following recipe for the preparation of homemade soap from the resin of *Picea abies* was documented in Lueta: 3 kg of fat or bacon, 1 kg of superalkali and resin, and 6–7 peeled potatoes are boiled for 1–2 h and then covered and allowed to stand until the following day.

All of the studied species are known in the study area as Christmas trees. Branches of *Picea abies* serve as decoration in front of the bride’s house on the wedding day (in Crăciunel). Needles of *Abies alba* were used on the surface of cheese for its conservation (Figure 3B).

The wood of the pine species was used for various tools and as timber, e.g., *Picea abies* for doors, boards, windows, and houses (in Lueta) and *Pinus nigra* for roofing (in Petreni). The wood of *Pinus sylvestris* was not recommended as timber because the resulting buildings would collapse in 2 years.

## 3. Discussion

The local names of selected pine species in Transylvania: ***fehérfenyő*** for *Abies alba*, ***veresfenyő*** for *Picea abies*, and ***lucfenyő*** for *Pinus sylvestris* were found to be similar to the earlier recorded names [51,53,58,110]. However, the majority of earlier works mention pines under the collective name of *fenyő* [51,53,85,86,98].

The ethnomedicinal use of *Abies alba* is well-documented in Transylvania and other European countries. Our recordings on this species show that it was used in the study area only to treat coughs and decayed teeth. However, we could not confirm, e.g., the use of resin of this species in the treatment of dermatological disorders [8,54,73,111,112,113].

Our results suggest that *Picea abies* was the most frequently used in the ethnomedicinal practice in the selected Transylvanian villages. We recorded some previously unknown uses of this species: cones for silicosis, burns, kidney diseases, and backache; needles for cardiac problems, thyroid glands, and cystitis; resin for burns; shoots for piles, paralysis, and as a sedative drug; and shoots or bark for varicose veins. Our results confirm the use of this species for respiratory diseases, dermatological disorders, and rheumatism, which was widely known from other studies in Transylvania [51,52,53,54,56,58,60,61,63,66,85,90] and other European countries [6,7,9,67,73,75,84,86,111,113,114,115,116]. The informants in our study area described several multiherbal remedies using different parts of *Picea abies*. Some of them, e.g., a seven-part ointment containing pine species resin, were previously documented in Transylvania by Miklóssy [85], but the plant source of resin was unidentified. The resin of *Picea abies* by itself for furuncles was documented in our study, while previous studies reported the use of resin combined with tallow, honey, wax, fat, and leaven [53,85].

We confirmed the use of the shoots of *Pinus nigra* in the treatment of respiratory diseases [91,111]. Furthermore, the use of the cones as a syrup for cold therapy and their resin as a poultice for dermatological disorders and dental problems is newly recorded in the study area.

Our records regarding *Pinus sylvestris* confirm the use of its different parts (cones, resin, and shoots) in the treatment of respiratory and dermatological diseases earlier reported in Transylvania [52,53,54,55,57,58,61,62,63,66] and other European countries [9,72,75,91,93,112,115].

Our study confirms the use of pine species in the ethnoveterinary practice. For example, the resin of *Pinus syvestris* is used in the study area to treat equine wounds [54,55], but we did not document the use of the plant as fodder reported earlier [107].

The studied pine species were also used for other purposes. Our informants shared with us their knowledge on several uses of *Picea abies* in the study area, which were mentioned in earlier surveys in Transylvania, but not known from other European countries related to the Carpathian Basin, e.g., the use of the bark for tanning processes of raw leather [81] and resin for homemade soap [102].

The resin of *Picea abies* and *Pinus sylvestris* was used for coating the violin bow, which is in accordance with the study by Pâques [108], who suggests that the resin of *Pinus sylvesteris* is still in use for colophony (rosin) in Romania.

The bark of *Pinus nigra* and *Pinus sylvestris* as a container for cottage cheese was known in Hungary, the Balkans, and Italy [47,98]. The use of the resin of *Pinus sylvestris* for soap was reported earlier by Rácz and Füzi [89]. The needles of *Abies alba* were used to preserve cheese, which was not mentioned in earlier studies. The use of the cones of *Pinus nigra* and *Pinus sylvestris* as tinder was not reported earlier. The wood of the pine species was used for various tools and as timber, e.g., in the case of *Picea abies* for doors, boards, windows, and houses and that of *Pinus nigra* for roofing. The same utilization was earlier reported from Transylvania [58,63,102,107] and other European countries [7].

All the studied species were known in the study area as Christmas trees, which was also mentioned in earlier studies [52,56,58,61,99,102,104,107], except in the case of *Pinus nigra*.

## 4. Materials and Methods

### 4.1. Study Area

The study was performed in the Harghita and Satu Mare counties in Transylvania (in the extended sense that includes Transylvania, Banat, Crișana, and Maramureș), a former part of Hungary, currently belonging to Romania [117]. The study area is surrounded by the Eastern and Southern Carpathian Mountains, and a large part is covered with coniferous forests [118]. *Abies alba* is mainly distributed in the Carpathians (e.g., Hargitha Mountains) as the dominant generalist species in coniferous forests [119], occurring at altitudes of (450) 600–1300 (1500) m [120]. *Picea abies* tends to form stands with other firs and beeches in montane and subalpine zones, at altitudes of (650) 800–1500 (1600) m, involving several cultivars as ornamentals. Pine species, such as *Pinus sylvestris* and *Pinus nigra,* grow naturally in higher regions of the Carpathians, where they have also been extensively planted [120].

**Figure 4:**
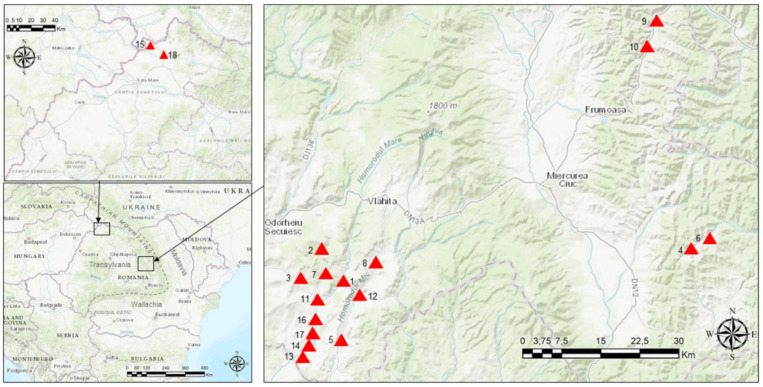
The study area in Transylvania (Romania). Numbers refer to the studied villages: (1) Aldea, (2) Călugăreni, (3) Chinușu, (4) Cinod, (5) Crăciunel, (6) Egershec, (7) Ghipeș, (8) Lueta, (9) Lunca de Jos, (10) Lunca de Sus, (11) Martiniş, (12) Mereşti, (13) Orășeni, (14) Petreni, (15) Porumbeşti, (16) Rareș, (17) Sânpaul, and (18) Turulung.

In the frame of the study, we selected 18 villages, where informants use the Hungarian language and dialects to facilitate communication (Table 4). The map of the study area was prepared by using the ArcGIS program (Figure 4).

The traditional knowledge of rural people working in agriculture and on farms is based on personal experience, on data passed on by their ancestors, and, in some cases, on other sources (e.g., books and media). The latter type of data was eliminated by using targeted questions about the origin of their knowledge; thus, only records of traditional knowledge are listed in this work.

Only 4 villages have a permanent pharmacy and facilities for human and veterinary medical service; 3 villages have only two of these three, while 11 villages in the study area have none of the three facilities.

### 4.2. Data Collection

The field study was conducted during the summer in the period 2007–2019. Before performing the interviews, consent was obtained from the participants, and ethical guidelines adopted by the International Society of Ethnobiology [121] were followed. The data gathered by semi-structured interviews were systematically arranged by collecting location and time, plant harvesting and storage method, administration, remedies, and treated disorders. Data documentation was carried out by voice recordings, handwritten notes, photographs of plants, drugs and remedies derived from them, and plant habitats.

The vernacular (local) name of pines, plant features, diseases, and original notes regarding the administration of the drugs are written in italics and bold based on folk terminology, while the names and terms corresponding to the official Hungarian and Romanian terminology were noted only in italics. The names of the disorders treated are in accordance with the orthography of the International Classification of Diseases (ICD-11).

During the ethnobotanical field surveys, all plants listed were documented according to the abovementioned aspects; however, this work summarizes only the records of the pine species *Abies alba*, *Picea abies*, *Pinus nigra*, and *Pinus sylvestris* identified by the botanist N. Papp. The names of identified plants follow the database of World Flora Online [122]. Voucher specimens of pine species (voucher specimens: PT04, PT07, PT11, PT12, PT16, PT17, PT19, and PT20) were deposited at the herbaria of the Department of Pharmacognosy, University of Pécs (7624 Pecs, Hungary).

### 4.3. Comparison to Literature

Our collected data on the pine species were compared to data gathered from earlier works performed in Transylvania (starting in the 16th century until 2021). Our literature research focused on the whole territory of Transylvania (including the villages where our study was performed) and sources from other countries related to the Carpathian Basin (Croatia, Hungary, Serbia, Slovakia, Slovenia, and Ukraine), collected from books, and mainly English-language references. The scientific articles associated with the studied pine species were selected from available databases, e.g., ScienceDirect, PubMed, and Google Scholar, using the following keywords: traditional, ethnobotanical, ethnomedicinal, and ethnopharmacological and filtered for the selected pine species. The results were illustrated as Venn diagrams.

## 5. Conclusions

The ethnobotanical and ethnomedicinal data recorded on the pine species confirm their current use in Transylvania. Despite several ethnobotanical studies being performed in this region, we registered several new and unique records, which highlight the current significance of ethnobotany in the study area. In comparison with previous studies, we could not confirm several data suggesting that the transmission of ethnomedicinal and ethnobotanical knowledge is on the decline.

## Figures and Tables

**Table 1:** Local names of the pine species in the study area.

Species	Local Name
*Abies alba* Mill.	***fehérfenyő*** ^3,4,6,8,10,11,14^ ***havasi fenyő*** ^11,14,17^ *jegenyefenyő* ^18^ *bradul alb* ^18^
*Picea abies* (L.) H. Karst	***veresfenyő*** ^5,8,12,14^ ***vörösfenyő*** ^4,6,8,14^ *fenyő*^1,2,7,11,13,15,16^ ***igazi fen yő*** ^11^ ***keresztfenyő*** ^12^ ***lucsika*** ^4,6^ *lucfenyő* ^10^
*Pinus nigra* J. F. Arnold	***luc*** ^5,11,14^ *fenyő* ^11,12,14^ ***luckfenyő*** ^11^ ***lucfenyő*** ^12,14^ ***lukszfenyő*** ^12^
*Pinus sylvestris* L.	***lucfenyő*** ^3,8,11,12,17^ ***lukszfenyő*** ^12^ *erdeifenyő* ^11,13^ *fenyő* ^4,6,9,11–13^ ***luc*** ^11^ ***lucsos*** ^11^ ***vörösfenyő*** ^3,10,11,13,14,17^

Folk names are written in italics and **bold**, while the official names are noted only in italics. Number superscripts refer to the studied villages: Aldea ^1^, Călugăreni ^2^, Chinușu ^3^, Cinod ^4^, Crăciunel ^5^, Egershec ^6^, Ghipeș ^7^, Lueta ^8^, Lunca de Jos ^9^, Lunca de Sus ^10^, Martiniş ^11^, Mereşti ^12^, Orășeni ^13^, Petreni ^14^, Porumbeşti ^15^, Rareș ^16^, Sânpaul ^17^, and Turulung ^18^.

**Table 2:** Ethnomedicinal data of the selected pine species in Transylvania compared to the literature data from the region and other Central and South European countries related to the Carpathian Basin.

Species	Part Used	Our Collected Data		Literature Data in Transylvania			Data from Other European Countries Related to the Carpathian Basin		
		Prepa- Ration	Use	Prepa- Ration	Use	Reference	Prepa- Ration	Use	Reference
***Abies alba* Mill.**	shoot	s ^11,14,18^	coughs ^11,14,18^	d, i	respiratory diseases	[52,61,63,66]	s	prevention of colds, coughs, respiratory diseases	[67]
		-	-	b	rheumatism	[54]	-	-	-
		-	-	-	-	-	d	digestive problems	[68,69]
	resin	p ^11^	decayed teeth ^11^	-	decayed teeth	[63]	-	-	-
		-	-	ch	teeth cleaning	[70]	-	-	-
		-	-	a	trepidancy	[54]	-	-	-
		-	-	with leaven	furuncles	[71]	-	-	-
		-	-	-	skin problems	[54]	-	wounds	[72,73]
		-	-	with fat	crackled udder of cows	[71]	-	-	-
		-	-	-	-	-	-	bruises	[74]
		-	-	-	-	-	p, to	arthritis	[75]
	bud	-	-	d	swollen cervical nodes	[54]	-	-	-
	bark	-	-	d	sore throat	[54]	-	-	-
	cone	-	-	d	stomachache, heart problems, toothache	[54]	-	-	-
	needle	-	-	d, s	respiratory diseases	[54]		catarrh of the respiratory tract	[76]
		-	-	c	liver pain	[54]	-	-	-
		-	-	-	-	-	-	neuralgia, rheumatism	[76]
		-	-	-	-	-	d	vitamin source	[77]
	wood	-	-	ash	wound	[54]	-	-	-
		-	-	-	-	-	dry and ground	cambium as flour for bread (food)	[77]
***Picea abies* (L.) H. Karst.**	bark	d ^8^	varicose veins ^8^	-	-	-	-	-	-
		-	-	d with milk	aphthous fever	[51]	-	-	-
		-	-		aphthae epizootic	[66]	-	-	-
		-	-	-	-	-	d, a, o	wounds arthritis	[75]
		-	-	-	-	-	externally	splints in case of bone fractures	[78]
	cone	s ^2,5,8,10,12,13^	asthma ^2,5,8,10,12,13^	i, d	coughs	[51,66,79,80,81,82]	a, d	productive coughs	[75]
			silicosis ^12^	-	-	-	-	-	-
			food ^8^	-	-	-	-	-	-
		d ^4,6,7,8^, with honey ^8,10,13^	burns ^8^	-	-	-	-	-	-
			coughs ^4,6,7,8,10,13^ dyspnoea (*fojlás*) ^8^ pneumonia ^8,13^ sore throat ^8^	-	-	-	-	-	-
			kidney diseases ^4,6^	-	-	-	-	-	-
		b ^8^	backache ^8^	-	-	-	-	-	-
		-	-	boiled with milk	aphthous fever	[79]	-	-	-
		-	-	d	aphtae epizooticae	[66]	-	-	-
		-	-	-	-	-	d	pyorrhoea	[83]
		-	-	-	-	-	ground	flour for bread (food)	[84]
	needle	d ^8^	cardiac problems ^8^	-	-	-	-	-	-
			thyroid glands ^8^	-	-	-	-	-	-
			cystitis ^8^	-	-	-	-	-	-
		b ^8^	rheumatism ^8^	-	-	-	a, o	arthritis	[75]
		-	-	-	-	-	eo	external use	[84]
		-	-	-	-	-	s	inflammation and pain to strengthen the immune system	[5]
		-	-	-	-	-	s	respiratory diseases	[5]
		-	-	-	-	-	d	vitamin source	[77]
		-	-	-	-	-	with sugar	marmalade and honey (food)	[84]
		-	-	-	-	-	a	beverage	[84]
	resin	d ^4,6,8,11^	burns ^4,6^	-	-	-	-	-	-
			chewed as food ^8,11^	-	white teeth	[53]	-	-	-
		o ^5,8^ p ^2,11,15^	wounds ^2,5,8,11,15^	-	wounds	[48,54,59,85]	itself, or with fat	wounds	[6,73,86]
		o ^5,8,15^ p ^1,2,7^	furuncles ^1,2,5,7,8,15^ backache ^1,2,5^	-	furuncles	[53,54,60,85]	-	furuncles	[6]
		p ^1,2,5^	toothache ^1,2,5^	-	decayed teeth, toothache	[53,57,58,63,87]	-	-	-
		-	-	o	abscesses	[53,54,58,63]	-	abscesses	[6]
		-	-	with honey	antibacterial	[88]	-	-	-
		-	-	b	burn, sedative	[48]	-	-	-
		-	-	-	plaster	[89]	-	-	-
	shoot	b ^8^	paralysis ^8^ piles ^8^ varicose veins ^8^ sedative ^8^	-	-	-	-	-	-
		d ^8^	rheumatism ^8^	b	rheumatism	[52,61,66]	b	rheumatism	[9,75]
		s ^3,5,8,10,11,12,14,18^ honey ^8,16^	asthma ^3,5,8,10,11^ coughs ^3,8,12,14,18^ sore throat ^16^ stomatitis ^8^	s	coughs, respiratory diseases	[51,53,56,58,63,66,90]	s, i	prevention of colds, coughs, respiratory diseases	[6,67,75,84]
		p ^8^	thyroid glands ^8^	-	-	-	-	-	-
		-	-	ch	gastric acid	[56,80]	d	digestive problems	[68]
		-	-	-	-	-	s	vitamin source	[77]
		-	-	-	-	-	-	arthritis	[75]
	wood	-	-	-	-	-	externally	splints in case of bone fractures	[78]
		-	-	-	-	-	dry and ground	cambium as flour for bread (food)	[77]
***Pinus nigra* J. F. Arnold**	cone	s ^12^	colds ^12^	-	-	-	-	-	-
	resin	p ^5,11,12,14^	furuncles ^12^ warts ^12^ wounds ^5,11,12,14^	-	-	-	-	-	-
			decayed teeth ^12^	-	-	-	-	-	-
		-	-	-	-	-	ritual herb	evil forces, against bad dreams, for health of family members and animals	[75]
	shoot	s ^11,12^ a ^11^	colds ^12^ coughs ^11,12^	-	-	-	i	inflammation of the respiratory system	[91]
		-	-	-	-	-	s	honey, food	[77,92]
	needle	-	-	-	-	-	i, s	respiratory diseases	[91]
***Pinus sylvestris* L.**	cone	s ^17^	colds ^17^	s, a, d, d with rusty bacon	respiratory diseases	[52,53,55,58,61,63,66]	-	-	-
		-	-	d	digestive problems	[53]	d	digestive problems	[66,68]
		-	-	b	rheumatism	[52,61]	-	-	
		-	-	d	digestive problems and respiratory disease in animals	[66]	-	-	-
		-	-	-	loss of appetite of pig	[52,53,61]	-	-	-
		-	-	-	-	-	d	pyorrhoea	[83]
	resin	p ^11–13,17^	furuncles ^12^ warts ^12^ wounds ^11,13,17^	with fat	furuncles, swelling, warts, wounds,	[53,54,57,62]	-	skin problems, wounds	[9,91,92,93]
			decayed teeth ^12,17^	-	decayed teeth	[58,63]	-	teeth cleaning	[94]
		-	-	-	rheumatism, impetigo	[95]	-	-	-
		-	-	o	insect repellent	[54]	-	-	-
							a	tuberculosis	[96]
	shoot	d ^13,17^ s ^3,8,9,11–14^	asthma ^3,11^ coughs ^3,8,9,11–14^ colds ^12,17^ tracheitis ^11^	d, s	respiratory diseases	[54,66]	i, d, s, in wine	respiratory diseases	[9,72,75,97]
		-	-	b	rheumatism	[54,66]	eo, i	rheumatism	[9]
		-	-	b	insomnia	[54,66]	-	-	-
		-	-	-	-	-	b, eo	antimicrobial, antiseptic	[9]
		-	-	-	-	-	eo	aromatherapy	[9]
		-	-	-	-	-	in wine	analgesic, epilepsy, strokes	[72]
		-	-	-	-	-	-	hemorrhoids, dysentery, as a diuretic	[93]
	bark	-	-	-	-	-	tonic or tar	dermatological problems	[9]
	needle	-	-	d	varicose veins	[95]	-	-	-
		-	-	-	plaster	[89]	-	-	-
		-	-	-	-	-	i, s	respiratory diseases	[5,91]
		-	-	-	-	-	s	rheumatism and gout	[5]
		-	-	-	-	-	d	vitamin source	[9]

Number superscripts refer to the studied villages: Aldea ^1^, Călugăreni ^2^, Chinușu ^3^, Cinod ^4^, Crăciunel ^5^, Egershec ^6^, Ghipeș ^7^, Lueta ^8^, Lunca de Jos ^9^, Lunca de Sus ^10^, Martiniş ^11^, Mereşti ^12^, Orășeni ^13^, Petreni ^14^, Porumbeşti ^15^, Rareș ^16^, Sânpaul ^17^, and Turulung ^18^. Abbreviations of preparations: a = alcoholic extract, as = ash, b = bath, c = compress, ch = chewing, d = decoction, eo = essential oil, i = infusion, j = jam, o = ointment, p = poultice, s = syrup, and to = topically.

**Table 3:** Other uses of the pine species in Transylvania compared to literature data from the region and other European countries related to the Carpathian Basin.

Species	Plant Part	Our Collected Data	Literature Data in Transylvania		Data from Other European Countries Related to the Carpathian Basin	
***Abies alba* Mill.**	bark	for shoe sole as tanning ^14^	-	-	-	-
		-	for holder of cottage cheese	[98]	-	-
	resin	-	-	-	wrapped in bark for scented room	[71]
	shoot	Christmas tree ^3,4,6,8,11,17,18^	Christmas tree	[56,58,82,99]	-	-
		-	for house of brides	[56,82]	-	-
		-	maypole	[79]	-	-
		-	symbol of a new house construction	[54]	-	-
		-	part of traditional wedding ceremony	[54]	-	-
	wood	-	tools	[53,63]	-	-
***Picea abies* (L.) H. Karst.**	bark	tanning ^8^	tanning	[100]	-	-
		-	dyeing	[101]	-	-
			fodder for sheep	[82]	-	-
	cone	-	dyeing	[100]	-	-
	needle	-	layered with hay as bed	[102]	-	-
	resin	epilation ^8^	-	-	-	-
		homemade soap ^8^	with fat for soap	[102]	-	-
		rosining violin bows ^8^	-	-	-	-
		-	chewing gum	[82,102,103]	-	-
		-	resin with splinters for fire	[103]		
		-	-	-	smoked resin for bees as a sedative	[86]
	shoot	Christmas tree ^8,12,17^	Christmas tree	[52,61,102]	-	-
		food ^11^	-	-	-	-
		incense in church ^12^	-	-	-	-
		ornament for house of brides ^5^	-	-	ornamental plant	[104]
		-	fodder for sheep	[82]	fodder	[104]
		-	-	-	ritual use	[104]
	wood	tools ^4,6^	tools	[58,63,82,102]	-	-
		furniture ^14^	furniture	[53,56,105]	-	-
			timber	[53,56,105]	-	-
			firewood	[52,61,102]	-	-
***Pinus nigra* J. F. Arnold**	bark	holder for cottage cheese ^12^	-	-	-	-
	cone	tinder ^12^	-	-	-	-
	resin	adhesive, dye additive ^12^				
	shoot	Christmas tree ^12^	-	-	-	-
		-	as flail for weaving loom	[106]	-	-
		-	-	-	timber	[91]
	wood	roof ^14^	-	-	-	-
		-	-	-	firewood	[91]
***Pinus sylvestris* L**.	bark	holder for cottage cheese ^4,6,12^	-	-	-	-
	cone	tinder ^9,12^	-	-	-	-
		-	Christmas decoration	[107]	-	-
	resin	rosining violin bows ^2^	rosining violin bows	[108]	-	-
		-	smoke and crock of burn resin for black dye	[109]	-	-
	shoot	Christmas tree ^12^	-	-	ornamental plant	[104]
	needle	-	soap	[89]	-	-
	wood	-	furniture	[58,63,82]	-	-
		-	tools	[58,63,82]	tools	[105]
		-	-	-	firewood	[91]
		-	-	-	construction material, timber	[91,104]

Number superscripts refere to the studied villages: Aldea ^1^, Călugăreni ^2^, Chinușu ^3^, Cinod ^4^, Crăciunel ^5^, Egershec ^6^, Ghipeș ^7^, Lueta ^8^, Lunca de Jos ^9^, Lunca de Sus ^10^, Martiniş ^11^, Mereşti ^12^, Orășeni ^13^, Petreni ^14^, Porumbeşti ^15^, Rareș ^16^, Sânpaul ^17^, and Turulung ^18^.

**Table 4:** Data on the study area in Transylvania (Romania).

Settlements	Latitude	Longitude	Altitude (m)	County	Informants/Inhabitants	Medical Service	Veterinary Practice	Pharmacy
Aldea	46°15′09″	25°26′06″	513	Harghita	15/362	-	-	-
Călugăreni	46°17′32″	25°24′04″	662	Harghita	15/52	-	-	-
Chinușu	46°15′34″	25°21′39″	588	Harghita	18/143	-	-	-
Cinod	46°18′14″	26°03′52″	980	Harghita	45/200	-	-	-
Crăciunel	46°11′00″	25°25′51″	530	Harghita	23/450	-	-	-
Egershec	46°18′13″	26°03′55″	980	Harghita	25/200	-	-	-
Ghipeș	46°15′52″	25°24′17″	576	Harghita	12/138	-	-	-
Lueta	46°16′27″	25°29′15″	610	Harghita	85/2900	-	+	+
Lunca de Jos	46°34′41″	25°59′43″	856	Harghita	32/1091	+	+	+
Lunca de Sus	46°31′44″	25°57′33″	908	Harghita	17/809	+	+	+
Mărtiniș	46°14′00″	25°23′00″	505	Harghita	21/570	+	+	+
Mereşti	46°13′59″	25°27′21″	558	Harghita	60/1319	+	-	+
Orășeni	46°09′50″	25°21′56″	490	Harghita	28/240	-	-	-
Petreni	46°10′37″	25°22′36″	490	Harghita	12/120	-	-	-
Porumbești	47°58′45′′	22°58′52′′	122	Satu Mare	12/1420	+	-	+
Rareș	46°12′27″	25°23′15″	490	Harghita	20/136	-	-	-
Sânpaul	46°11′29″	25°22′56″	490	Harghita	15/494	-	-	-
Turulung	47°55′00″	23°05′00″	132	Satu Mare	60/3500	+	+	+

Abbreviations: + available, - not available

## Data Availability

The data presented in this study are available on request from the corresponding author.
